# Entrepreneurship Education and Health-Stress Analysis of College Teachers and Students Using Backpropagation Neural Network Model

**DOI:** 10.3389/fpsyg.2022.783188

**Published:** 2022-03-17

**Authors:** Leiming Fu, Qi Cheng

**Affiliations:** ^1^School of Information Management, Nanjing Agricultural University, Nanjing, China; ^2^School of Marxism, Anhui University, Hefei, China

**Keywords:** Marxism, BP neural network, college teachers and students, entrepreneurship education, health and stress

## Abstract

The purpose is to solve the problem of college students’ employment difficulties. It is the development trend of the times to master the basic psychological pressure state of students and analyze students’ problems by using modern technology and science. First, based on Marxist theory, the theory of entrepreneurship education and the characteristics of teachers and students in colleges are expounded, and the principle and algorithms of Backpropagation Neural Network (BPNN) are introduced. Second, from the perspective of entrepreneurship education and mental health factors of college students, the sample set of the BPNN model is collected using a Questionnaire Survey (QS). Then, the sample set is normalized to analyze the current college entrepreneurship education and the health and stress of college students. The results show that the comprehensive BPNN output of entrepreneurship education is 0.726, indicating that entrepreneurship education in colleges is at a moderate level. The proposed BPNN model can perform better than the traditional prediction methods in predicting college students’ mental health, and the data fitting results are satisfactory. Overall, college students’ entrepreneurship education should be improved, and schools should take more incentives to help adjust college students’ mentality.

## Introduction

The modern world is undergoing a profound change, and education has become the key to a nation’s development. With rapid technological development and international cooperation, competition has intensified, and economic globalization is accelerating (Qian et al., 2018; Lin and Lin, 2021). Entrepreneurship education is the foundation of national rejuvenation and social development. China is going through a transitional period of reform and opening up, in which the economic reform is deepening. The great goal of socialist modernization construction should be realized (Rogoza et al., 2018; Chen, 2019). National construction and ecology, such as economic construction, political construction, cultural construction, and the control of environmental pollution and resource consumption need various professional talents. Therefore, a comprehensive and scientific educational system is needed to improve the overall quality of the national population and cultivate a group of high-quality and innovative talents, which is also the ardent expectation and demand of the people (Wu W. et al., 2019; Wu and Song, 2019). China has attached great importance to education and personnel training since ancient times. Today, China’s educational achievements are related to the speed of China’s development, the level of China’s socialist modernization, and whether the great Chinese nation can stand proud in the world again (Wu and Wu, 2017; Wu W. et al., 2020). Talent is the key to China’s future development and the great rejuvenation of the Chinese nation, (Wu Y. J. et al., 2020; Yuan and Wu, 2020). Meanwhile, China’s future success lies in education, and every Chinese is responsible for revitalizing education, which is why education is put in a critical position to ensure the successful implementation of the educational policies of the Communist Party of China (CPC). The four modernizations of China and the construction of a prosperous society require constant exploration, advancement, and innovation in talent cultivation.

In the past few years, with the continuous social progress, college students have shown more and more mental health problems, which are getting serious. Many research reports emphasize that the proportion of college students with mental health problems is very high, especially in the dimensions of compulsion, anxiety, depression, interpersonal sensitivity, and lack of self-confidence. Although much attention has been paid to the mental health education of college students and the medical development is speeding up, there are still multiple problems (Antal et al., 2017; Barba and Atienza, 2018). College students’ mental health is affected by multiple factors, each of which is inseparable. Hence, the prediction of college student’s mental health is a non-linear problem. In recent years, as a new type of intelligent information system, Artificial Neural Network (ANN) technology is developed from the biological Neural Network (NN) (Fellnhofer and Kraus, 2017; Mets et al., 2017). In practical application, NN technology solves many complex causality problems, which can be used for associative memory, non-linear mapping, classification, recognition, and intelligent operations, such as knowledge processing (Kirkley, 2017; Bischoff et al., 2018). Previous studies did not apply NN’s powerful prediction and analysis ability to mental health prediction. A more practical method is urgently needed to analyze and predict mental health more accurately. Here, an NN-based prediction algorithm is utilized to predict students’ mental health problems.

The college teachers and students are taken as the research object based on the above research content. The Questionnaire Survey (QS) combined model test is adopted to analyze the current situation of college teachers and students’ entrepreneurial consciousness. From the perspective of innovation, the proposed Backpropagation Neural Network (BPNN) model can perform better than the traditional prediction methods in predicting college students’ mental health, and the data fitting results are satisfactory. The health and pressure of college students are discussed to provide some reference for college students’ entrepreneurial awareness and mentality adjustment, which is of great significance for promoting college students’ employment.

## Neural Network and Entrepreneurship Education of College Teachers and Students

### Entrepreneurship Education

China’s innovation and entrepreneurship education have a late start compared with foreign countries. However, innovation and entrepreneurship education for college students is rapidly catching up and has aroused great attention from all social spheres, and in-depth exploration and related research on entrepreneurship education is being conducted comprehensively. China’s innovation and entrepreneurship research should be further deepened, systematized, and constantly improved based on China’s actual situation (Gedeon, 2017; Fellnhofer, 2018). Some researchers believe that the reform of curriculum system, teaching content and methods, and the development of extracurricular practical activities can effectively cultivate students’ awareness of innovation and entrepreneurship. Wu Y. C. J. et al. (2019) proposed a classroom response system to study college students’ entrepreneurial experience to improve their awareness and ability in entrepreneurship (Wu Y. C. J. et al., 2019). Entrepreneurship education is an educational practice to cultivate students’ entrepreneurial spirit, knowledge, and other comprehensive qualities and promote students’ entrepreneurship and innovation. Some scholars argue that entrepreneurship education is a new concept and pattern of employment education, emphasizing students’ entrepreneurial awareness and ability (Fox et al., 2018; Sánchez et al., 2020). In short, the purpose of entrepreneurship education is to cultivate students’ comprehensive qualities and abilities for entrepreneurship. Some researchers suggest that the general purpose of entrepreneurship education is to improve students’ entrepreneurial awareness and cultivate their entrepreneurial quality. Specifically, entrepreneurship education is an education behavior to cultivate independent entrepreneurship knowledge, quality, and skills. Broadly, entrepreneurship education aims to cultivate people with entrepreneurial qualities. Some scholars point out that entrepreneurship education can specifically refer to behavior that cultivates students with the social and professional skills to become an entrepreneur from a job seeker. More broadly, entrepreneurship education can improve students’ overall quality. During entrepreneurship, students should learn from the curriculum system to foster themselves with the creative and innovative spirit, entrepreneurial consciousness, stable mentality, and decision-making mentalities. Zhou and Wu (2018) emphasized the importance of cultivating entrepreneurial awareness. Here, entrepreneurship education is defined both in a broad and narrow sense. Emphasis is put on spirit, vocational training, and education process in the broad sense definition. In the narrow sense, entrepreneurship education is defined as vocational education to encourage students to start a business successfully (Zhou and Wu, 2018).

## Characteristics of College Teachers and Students

College students are believed to be highly intelligent and outstanding youth representatives. Their mental health significantly influences their growth and success and the great rejuvenation of the Chinese nation and socialist modernization. College students are at the age of critical transitional period to physical and mental maturity. Without being properly treated, they may face multiple problems from their emotions and society, leading to more serious psychological problems, such as depression and anxiety. It greatly hinders the education of college students (Dash, 2017; Garcia et al., 2019). For example, it is not uncommon to find college students with emotional disorders. In most colleges, a specific number of students suffer from various mental problems with different degrees, which seriously affect their normal life and study. Additionally, according to domestic and international research, students with neuropsychiatric problems account for the most in college dropouts, so the mental health problems of contemporary students cannot be ignored.

With the increasing influence of college students’ mental health problems, psychological education has become the center of social concerns. Following the collection of college students’ mental health factors, these factors are classified into four categories.

### School Factors

The first is the ability to adapt to the environment, and the change of living environment is the basis of psychological change. The change of living environment is reflected in the change of collective middle school living environment to independent college life. It requires self-reliance and spiritual independence based on self-understanding, without which college students may suffer loneliness or even mental health problems. The second is the learning pressure, partly from an unclear understanding of their major. Meanwhile, the learning pressure comes from accepting new and more difficult knowledge structure that is completely different from those of middle school. Interestingly, college life may differ greatly from what many students have expected in their middle schools. With a heavier burden of learning, students are under long-term excessive mental stress. Additionally, examinations also bring great pressure to students. These factors all can contribute to various mental health problems and emotional tension. College students are young and energetic, and they long for friendships and romantic relationships. However, the hysteretic psychophysiological maturity usually leads to deviation from expectations, emotional pressure, or even mental health problems without proper management.

### Individual Factors

College students are ambitious for success and eager for knowledge. However, for practical reasons, many students have chosen a different major from their initial will, resulting in a negative psychological effect that sometimes causes a resistance emotion toward professional courses and then leads to mental health problems. Meanwhile, interpersonal pressure in college life gets intenser than that in middle schools. Psychologist Ding ye believed that the most important thing in human psychological adaptation was interpersonal adaptation. The main reason for human psychological diseases is the imbalance of interpersonal relationships. Some unavoidable interpersonal relationships will lead to various physical and mental symptoms, and if the interpersonal relationship stops, the symptoms will disappear immediately. Generally, interpersonal relationships, will continue to generate psychological pressure if not well-managed. Therefore, it is also an essential factor affecting college students’ mental health. Students with interpersonal problems often have bad relationships. Classmate relationship is also the most common form of interpersonal relationship, which easily produces contradictions. Research shows that 36% of college students’ mental health problems are related to the classmate relationship.

### Social Factors

First, social development pressure increases. As social development accelerates, fierce competition has been brought along with fast-paced life and labor intensity industries. Apart from the negative impact of the market economy, work pressure is getting increasingly heavy, and many people get psychological problems, such as depression and anxiety, which is no exception for college students. After reform and opening up, colleges provide more opportunities for students, but, meanwhile, various social problems are rampant, so college students feel many psychological contradictions and even get cynical about society. Some new college students’ personality weaknesses gradually emerge during this transitional social period. Next, college life and education are critical to forming students’ three outlooks (world outlook, view of value, outlook on life). Yet, with the rapid development of the information age, college students are faced with huge amounts of miscellaneous information that can be favorable or dangerous to their personality and mental health development based on personal choices. Hence, this may also contribute to college students’ psychological problems without correct guidance.

### Family Factors

Family factors are the most straightforward pressure that students get, partly from parents’ high expectations for their children. In China, the money and time parents spend on their children are tremendous, and some would like to see their children fulfill their parents’ wishes and impart children with all kinds of knowledge. Most students are willing to work hard because they know what’ on their shoulders. It increases the psychological burden of college students, which also contributes to students’ mental health problems. Meanwhile, parental behavior has an impact on college students’ pressure. It has been argued that parents are the best teachers for children, and their behaviors influence children, especially their personality formation. Therefore, the way parents raise and educate their children directly affects children’s behavior and psychology. A democratic and equal rather than an autocratic family environment can help develop students’ psychological health and alleviate pressure.

Abu-Tineh and Sadiq (2018) studied the characteristics of effective professional development and effective professional development model perceived by school teachers in the state of Qatar. The results show that school teachers in Qatar believe that the proposed characteristics of effective professional development are very effective. In addition, they think that the guidance model is the most effective and healthy professional development model. McCallen and Johnson (2020) pointed out that students’ success in public higher education is an indispensable part of modern social equity in the United States. The research shows that the multidisciplinary theoretical framework is adopted to consider the impact of social capital on the success of the first generation of college students in large public city colleges, and put the proximal and structural characteristics of shaping the opportunities of underrepresented students in the context. Machingambi (2014) believed that globalization had imposed values and spirit on the higher education system, intensifying educational inequality and social disharmony. Marxist theoretical methods are adopted to reflect and analyze the impact of globalization on the practice and process of higher education. In particular, it explores how the forces of globalization limit access, equity, capital and national culture.

## Neural Network

**FIGURE 1:**
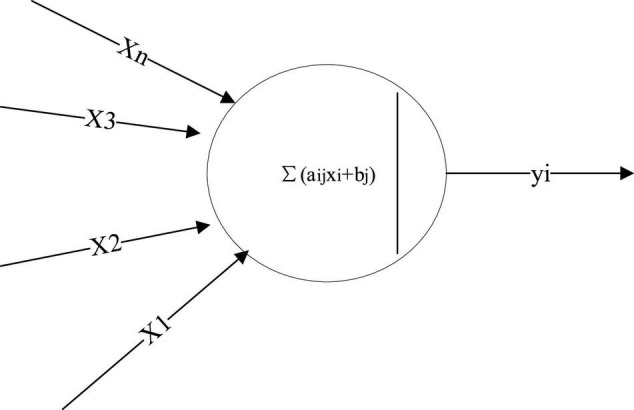
Structure of a neuron.

(1) ANN. ANN is a non-linear adaptive dynamic system composed of large numbers of neurons. The system has strong adaptability and autonomous learning ability, and it is non-linear and non-local (Muralidhar et al., 2017; Huang et al., 2019). Based on modern neuroscience, a new machine is designed here through the simulation of the brain NN to process the received information and memory information. The basic processing unit of ANN is a non-linear device with multiple inputs and a single output. Figure 1 displays its structure.

In Figure 1, Xn represents the input signal, and y_*i*_ denotes the output signal. In the model, the middle transformation equation reads:


(1) $$y_{i}=f\,\left(\sum a_{i\,j}\,x_{i}+b_{j}+c_{j}\right)$$

**FIGURE 2:**
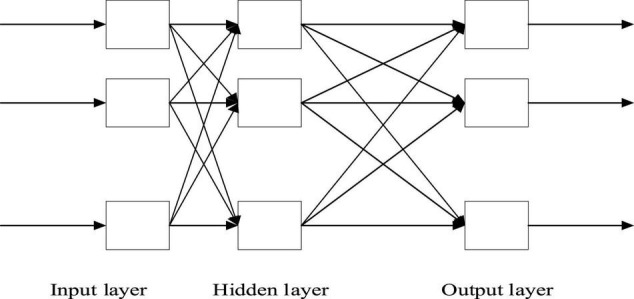
BPNN structure.

**FIGURE 3:**
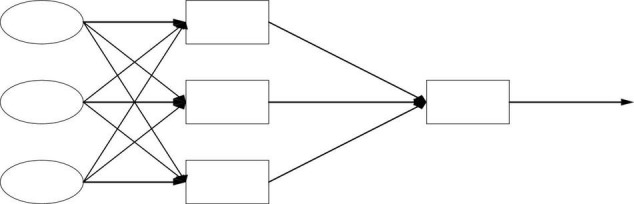
Feedforward network structure.

**FIGURE 4:**
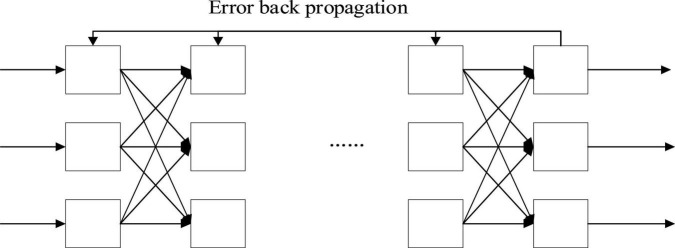
Structure of multilayer error backpropagation NN.

(2) BPNN model. The three-layer BPNN model is the most common model, which includes the input layer, hidden layer, and output layer from left to right. The data are input from the leftmost input layer and then output from the rightmost output layer. They are fully connected between the layers (An et al., 2017; Sufiyah et al., 2018). Essentially, BPNN is a non-linear input-output mapping. The network input is considered the independent variable of the non-linear function, and the network output (prediction) value is the dependent variable of the non-linear function. Given the input and output nodes: *a* and *b*, respectively, BPNN represents the function mapping relationship *a-b*, as shown in Figures 2–4.

The basic principle of the BP algorithm is to divide the learning process into two processes: the forward propagation of signal and the backward propagation of error. Each neuron in the input layer receives external input information and transmits it to each neuron in the hidden layer. The hidden layer is responsible for internal information processing and information conversion. According to the needs of information change, one or more hidden layers are designed. After being processed through each hidden layer, the information is transmitted to the output layer, which is the process of outward propagation. When the actual output of the output layer does not match the expected output, error backpropagation is triggered (Mary et al., 2018; Herlosky and Tran, 2019). The error is transmitted and distributed among the hidden layer, the output layer, and then the input layer. Therefore, the neurons in each layer of the error signal can be used as the basis to correct the reconnection of each layer. The signal circulates forward, while the error circulates backward during propagation. The weight of each layer is adjusted until the output error of the ANN is reduced to an acceptable threshold or the previously set number of learning times is reached.

Using the error feedback algorithm, BPNN can learn and store multiple mapping relationships between input patterns and output patterns without revealing the mathematical equations describing these mapping relationships in advance (Chun, 2017; Zeng et al., 2017). The learning rule is to use the fastest descent method and feedback propagation to constantly adjust the weight and threshold of the network to produce the least sum of squares of network errors. BPNN consists of three neural layers: the input, output, and hidden layers. There is only one input layer and one output layer. The hidden layer is manually defined and can be one or more layers. The most extensive and simple BPNN contains only one hidden layer, where each layer contains one or more nodes that are fully interconnected through layers. In this way, each node is fully connected with the upper layer nodes of each layer but does not belong to the same layer between nodes, that is, there is no feedback between nodes.

(3) BP learning algorithm. *a*_*i*_ represents the BP input layer, *b*_*j*_ denotes the middle hidden layer, and *c*_*k*_ indicates the output layer. *f*_*ij*_ stands for the connection weight between the input layer and the hidden layer. *g*_*jk*_ means the connection weight between the hidden layer and the output layer, *d*_*l*_ and *e*_*m*_ refer to the thresholds of the output layer and the hidden layer, respectively, and *h*_*n*_ is the expected output of the output layer.

In the BPNN model, the *net* represents the neuron. Its expression reads:


(2) $$n\,e\,t=\sum_{i=1}^{n}g_{i}\,a_{i}-e$$

The activation function of BPNN reads:


(3) $$g\,\left(a\right)={\left(1+e^{-k\,a}\right)}^{-1}$$

The derivative function of the BPNN activation function reads:


(4) $$g^{\prime }\,\left(a\right)=g\,\left(a\right)\,\left(1-g\,\left(a\right)\right)$$

The principle of BPNN is to divide the learning process into two processes: the forward propagation of signal and the backward propagation of error.

The signal forward propagation of BPNN:

From the input layer to the hidden layer, the output reads:


(5) $$b_{j}=g\,\left(n\,e\,t_{j}\right)=g\,\left(\sum_{i=1}^{n}p_{i\,j}\,a_{i}-e_{j}\right)$$

For the convenience of calculation, let


(6) $$n\,e\,t=n\,e\,t_{j}=\sum_{i=0}^{n}p_{i\,j}\,a_{i}$$

From the hidden layer to the output layer, the output reads:


(7) $$c_{k}=f\,\left(n\,e\,t_{k}\right)=f\,\left(\sum_{i=1}^{n}g_{i\,k}\,b_{j}-d_{l}\right)$$

For the convenience of calculation, let


(8) $$n\,e\,t=n\,e\,t_{j}=\sum_{j=0}^{n}g_{i\,j}\,b_{i}$$

The error backpropagation of BPNN:

The error function reads:


(9) $$E=\frac{1}{2}\,\sum_{k=1}^{n}e_{k}^{2}$$

Error function of hidden layer reads:


(10) $$E=\frac{1}{2}\sum_{k=1}^{n}[h_{n}-f{\left(\sum_{j=0}^{n}g_{i\,j}b_{j}\right]}^{2}$$

The error function of the input layer reads:


(11) $$E=\frac{1}{2}\sum_{k=1}^{n}[h_{n}-f(\sum_{j=0}^{n}g_{i\,j}p{\left(\sum_{j=0}^{n}p_{i\,j}a_{i}\right]}^{2}$$

Equation 8 shows that the error function is the function of the weight of the connection between the input layer and the hidden layer *f*_*ij*_ and the weight of the connection between the hidden layer and the output layer *g*_*jk*_. Then, the error function *e* can be reduced by altering the connection weight. The ultimate goal is to continuously reduce the weight, and Eqs. 12, 13 are obtained.


(12) $$\Delta \,P_{i\,j}=-\eta \,\frac{\partial E}{\partial p_{i\,j}}$$

(13) $$\Delta \,g_{j\,k}=-\eta \,\frac{\partial E}{\partial g_{j\,k}}$$

The learning rate of BPNN is reflected in Eqs. 12, 13.

Next, the error signal is defined. Then, the corresponding weights and thresholds are adjusted to show the same gradient changes.


(14) $$\delta_{j\,k}=-\sum_{k=1}^{k}\left(h_{k}-c_{k}\right)\,c_{k}\,\left(1-c_{k}\right)$$

(15) $$\delta_{j\,k}=\delta_{j\,k}\,g_{j\,k}\,b_{j}\,\left(1-b_{j}\right)$$

The equation of weight adjustment of the hidden layer reads:


(16) $$\Delta \,p_{i\,j}=\eta \,\delta_{i\,j}\,a_{i}$$

The equation for weight adjustment of output layer reads:


(17) $$\Delta \,g_{j\,k}=\eta \,\delta_{j\,k}\,b_{i}$$

The equation of threshold adjustment reads:


(18) $$\Delta \,d_{l}=-\eta \,\delta_{i\,j}$$

(19) $$\Delta \,e_{m}=-\eta \,\delta_{j\,k}$$

**FIGURE 5:**
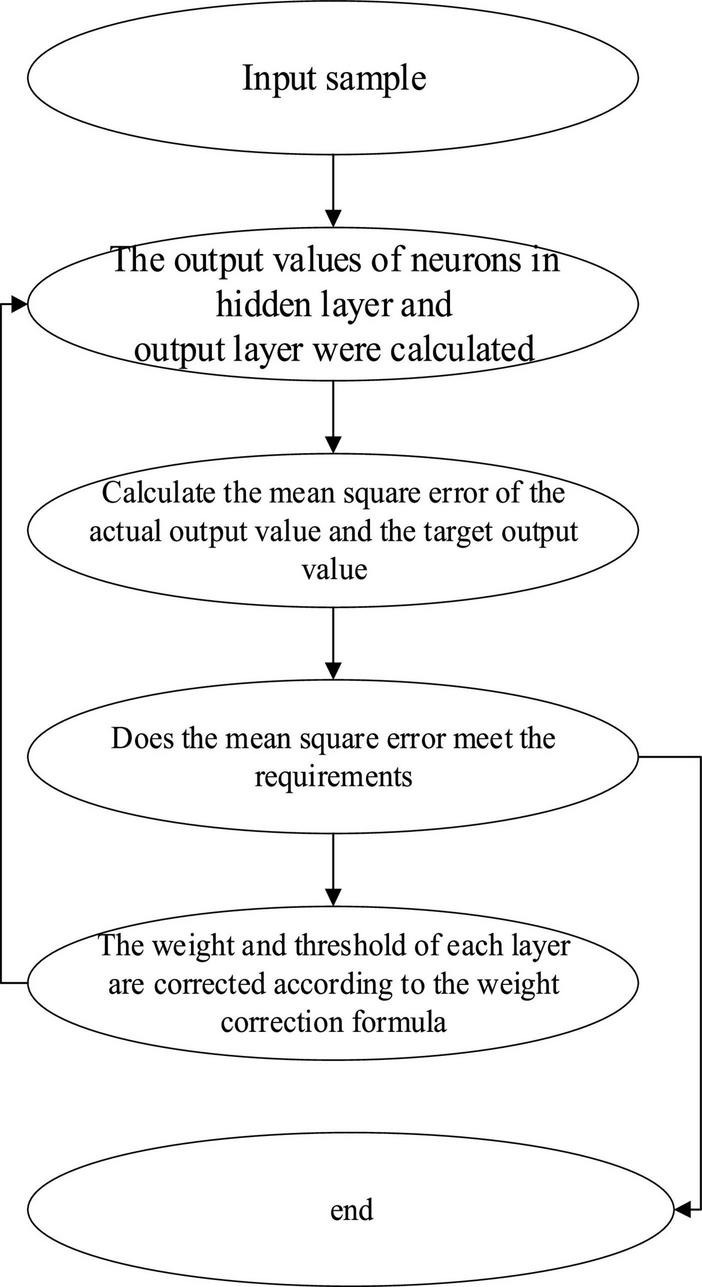
Operation flowchart of BPNN.

(4) NN operation process. Figure 5 is the flow chart of BPNN.

## Backpropagation Neural Network Model Design and Method

Questionnaire Survey is designed in this chapter, and then the neural network is modeled. The modeling of neural networks mainly includes four objectives. First, it is to establish sample set data; second, it is to design the model; third, it is to select the network learning parameters and data preprocessing; fourth, it is to conduct network training.

## Design of Questionnaire Survey

According to the above analysis of entrepreneurship education for college teachers and students, the QS will be designed from the student, society, school, and government dimensions. The student dimension includes scientific research ability, entrepreneurship rate, innovative practice, and innovative achievements. The society dimension includes social atmosphere, corporate groups, and social reputation. The school dimension includes curriculum system, school running idea, teaching staff, practice basis, and campus culture. The government dimension includes investment, management systems, and policy optimization. Based on the above analysis of the factors of contemporary college students’ mental health problems, emotional pressure, the QS also involves future employee pressure, social pressure, learning pressure, economic pressure, environmental pressure, parents’ expectations, family influence, occupational emotion, and interpersonal relationship pressure. Finally, the QS is designed. Table 1 presents the key questions set.

**TABLE 1:** Key questions of the QS.

Content	Question
Health and stress	Q1 With the development of society, the pressure of competition is increasing. It is not easy for college students to find a desirable job. What do you think of the pressure of your future life?
	Q2 With the development of the Internet, great changes have taken place in all social spheres, especially, in information. What do you think of this?
	Q3 With the reform of the national education system and the increase in living expenses, what do you think of the pressure on life?
Entrepreneurship education	Q4 How much do you know about the entrepreneurship education course in your school?
	Q5 Do your school adopts ways to encourage teachers and students to carry out innovation and entrepreneurship education?
	Q6 How is your entrepreneurship education competition going?

## Neural Network Modeling

**FIGURE 6:**
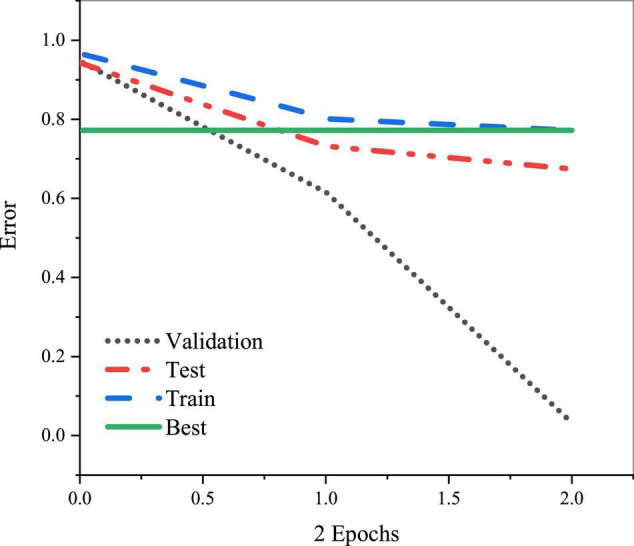
Training results of entrepreneurship education based on BPNN.

(1) The establishment of the sample set data. For BPNN, data preparation is critical. Good sets of data can quickly balance the convergence of the network and approach the output result. On the contrary, the network cannot achieve the desired effect with bad datasets through parameter adjustment. Here, the input data are collected from the designed QS. Totally, 120 QSs are distributed, 120 are collected, and 110 are valid. In the QS design, A, B, C, D, and E represent 0.5, 0.4, 0.3, 0.2, and 0.1 scores, respectively.
(2) Model design. First, the design of the input and output layer: the input and output layer mainly receives the input data and the final output results. Generally, the number of the network input layer equals the number of input problems, and the number of neurons in the output layer equals the number of the output problems. The number of neurons in the input and output layers is 10 and 1, respectively. Second, the design of the hidden layer. BPNN has a hidden layer, which can deal with more complex problems. Usually, hidden layers are one or two layers. The number of neurons can be selected flexibly according to the actual situation for optimal network precision. The proposed network model consists of a hidden layer and 15 neurons. The NN data should be pretreated to improve the efficiency and speed of NN formation. The preprocessing method of MATLAB includes the normalization and analysis of the main components, in which normalization is usually used, that is, the input and output data are mapped to the range of [−1,1], and then mapped to the original data range after training. Meanwhile, the data must be disordered and classified. Among the 110 input data, 25% are test data, 25% are change data, and the remaining 50% are used for normal input and training. It is the difference between the neural network model designed and the general neural network.
(3) The selection of network learning parameters and data preprocessing. Generally, NN must learn the determination of parameters and learning rate, so the initialization method is significant for the network convergence and training time. Besides, the output value of each neuron is expected to be close to zero after the initial weighting, thereby ensuring that the weight of each neuron is adjusted at the place where the type activation function changes the most. Therefore, the initial weight is usually a random number between 0 and 1. The next is the learning rate selection. Learning rate, also known as the phase, determines each training cycle’s weight and closure value. A too-high learning rate may lead to system instability. Comparatively, a too-low learning rate may extend the learning time, resulting in slow network convergence. However, the network error does not exceed its surface depression and approximates the minimum error. Therefore, commonly, a lower learning rate is chosen to ensure the stability of the system.
(4) Network training. First, the init function is used for initialization, and the target vector T and P are input according to the dataset. The network prediction model is created through the newff. Figure 6 is the training result.

**FIGURE 7:**
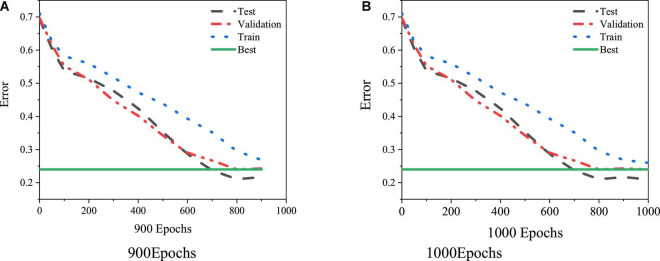
BPNN error curve. **(A)** 900 Epochs. **(B)** 1,000 Epochs.

The function newff is used for the parameter setting of the BPNN, and then the training function can train the BPNN model. According to the practical experience, the training time and the learning speed are set as 9,000 and 0.02, respectively. After model training, the change of error is obtained, as shown in Figure 7.

**FIGURE 8:**
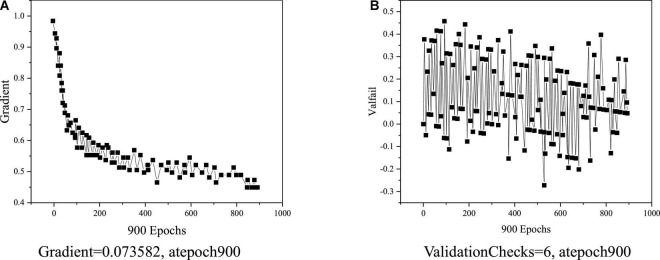
BPNN training status. **(A)** Gradient = 0.073582, atepoch 900. **(B)** ValidationChecks = 6, atepoch 900.

Figure 7 illustrates that the model converges well, and it can meet the requirements of this experiment when the number of iterations reaches 900. Figure 8 displays the training state.

**FIGURE 9:**
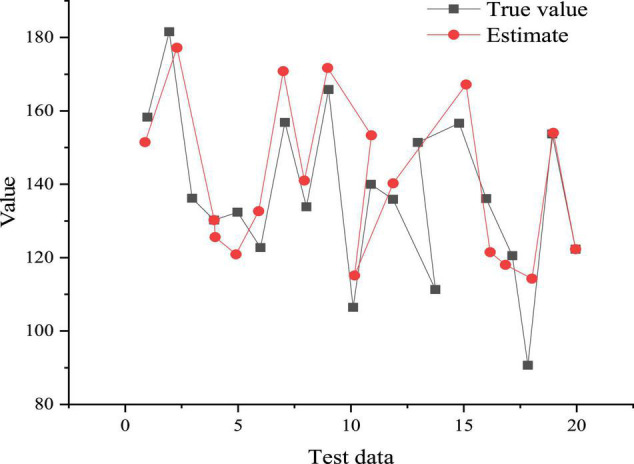
Comparison of estimated value and output value.

(5) Network simulation test. The function sim can simulate the trained network, and then the simulation results are de-normalized and compared with the original data. Figure 9 displays the comparison result of the estimated value with the real value.

Figure 9 shows that the estimated value is close to the real value. Hence, after training, the BPNN model can better predict the health and stress of college teachers and students in entrepreneurship education. To sum up, after 900 iterations of training, the average variance value of the BPNN model is only 0.0303, so the predictability of the proposed BPNN model is better.

## Analysis of the Results of Entrepreneurship Education and Health Stress Test

Through the relevant theories of Marxism and entrepreneurship education, the BPNN model is put forward based on the analysis of the characteristics of college teachers and students. On this basis, the entrepreneurial consciousness of college teachers and students and the factors affecting their development are analyzed through QS method and model test.

**FIGURE 10:**
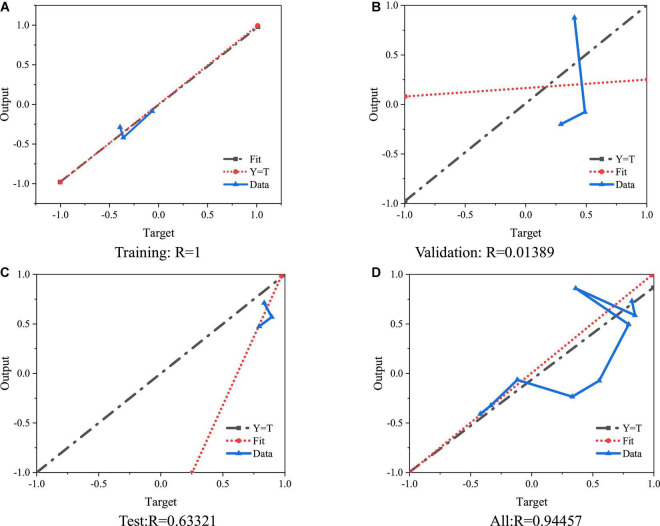
Training error based on BPNN. **(A)** Training: *R* = 1. **(B)** Validation: *R* = 0.01389. **(C)** Test:*R* = 0.63321. **(D)** All:*R* = 0.94457.

The collected data of entrepreneurship education are normalized, and then the dataset is simulated with the function sim. Figure 10 presents the results.

The predicted value of the neural network model designed can be obtained as 0.726 from the curve distribution in Figure 10, which shows that the entrepreneurship education of college students is at the medium level and needs to be further improved. Therefore, colleges should start from the above four aspects, focus on entrepreneurship education, cultivate talents, and improve employment. The prediction results also have strong feasibility, and the model has a high prediction value because the accuracy of the designed prediction model is guaranteed.

**FIGURE 11:**
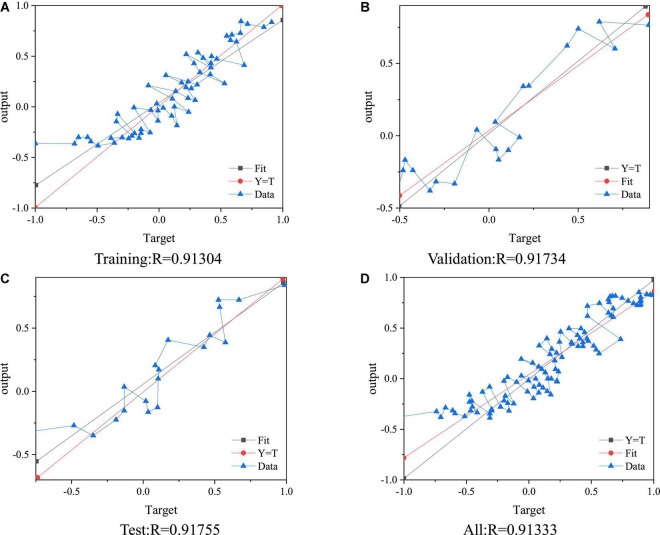
BPNN linear regression processing. **(A)** Training:*R* = 0.91304. **(B)** Validation:*R* = 0.91734. **(C)** Test:*R* = 0.91755. **(D)** All:*R* = 0.91333.

The dataset of college students’ health stress is processed by linear regression. Figure 11 displays the results.

Figure 11 suggests that the proposed BPNN model performs well for the sample set’s training, verification, and test mode.

## Conclusion

Under the pressure of employment, the mental health of college students has attracted widespread attention from all walks of life. Here, the BPNN model is proposed based on Marxist and entrepreneurship education-related theories and the analysis of the characteristics of college teachers and students. The sample dataset of the neural network prediction model is from the questionnaire survey, which ensures the effectiveness of the data. The data are further optimized by using the preprocessing method of MATLAB, so that the neural network can better analyze and process the data. Then, the QS method and model test are used. The entrepreneurial awareness of college teachers and students and the factors affecting their development are analyzed. The evaluation of the mental health of college students is discussed. The analysis of the proposed BPNN model indicates that the entrepreneurial awareness of college teachers and students should be strengthened, and the students with mental health problems can well be predicted, thereby formulating corresponding measures to help students grow and study healthily. In college teaching and learning processes, stress and health analysis is a hot topic in relevant research fields. In the study of the correlation between health belief and physique and stress adaptation of college teachers and students, scholars should know the impact of health belief on stress adaptation of college teachers and students and the impact of psychological elasticity on stress adaptation. The reason is that one of the most important factors of individual adaptation to stress is psychological elasticity. Only through the dual guarantee of mental health and physical health can the grass-roots people overcome the pressure with good mentality and physical quality in the face of various pressures generated by the surrounding environment to provide a strong driving force for work and life. Due to the influence of the actual experimental conditions, the selected research samples are limited, which is not enough to cover all aspects. The samples will be expanded in the later research, and more factors will be considered for entrepreneurship education, college students and teachers, and employment.

## Data Availability Statement

The raw data supporting the conclusions of this article will be made available by the authors, without undue reservation.

## Ethics Statement

The studies involving human participants were reviewed and approved by the Anhui University Ethics Committee. The patients/participants provided their written informed consent to participate in this study. Written informed consent was obtained from the individual(s) for the publication of any potentially identifiable images or data included in this article.

## Author Contributions

Both authors listed have made a substantial, direct, and intellectual contribution to the work, and approved it for publication.

## Conflict of Interest

The authors declare that the research was conducted in the absence of any commercial or financial relationships that could be construed as a potential conflict of interest.

## Publisher’s Note

All claims expressed in this article are solely those of the authors and do not necessarily represent those of their affiliated organizations, or those of the publisher, the editors and the reviewers. Any product that may be evaluated in this article, or claim that may be made by its manufacturer, is not guaranteed or endorsed by the publisher.
